# Impaired Antibody Response to Influenza Vaccine in HIV-Infected and Uninfected Aging Women Is Associated with Immune Activation and Inflammation

**DOI:** 10.1371/journal.pone.0079816

**Published:** 2013-11-13

**Authors:** Anita Parmigiani, Maria L. Alcaide, Ricardo Freguja, Suresh Pallikkuth, Daniela Frasca, Margaret A. Fischl, Savita Pahwa

**Affiliations:** 1 Department of Microbiology and Immunology, University of Miami Miller School of Medicine, Miami, Florida, United States of America; 2 Division of Infectious Diseases, University of Miami Miller School of Medicine, Miami, Florida, United States of America; 3 Department of Surgery, Oncology and Gastroenterology, Oncology and Immunology Section, Unit of Viral Oncology and AIDS Reference Centre, University of Padua, Padua, Italy; 4 UM AIDS Clinical Research Unit, Division of Infectious Diseases, University of Miami Miller School of Medicine, Miami, Florida, United States of America; Centers for Disease Control and Prevention, United States of America

## Abstract

**Background:**

Aging and HIV infection are independently associated with excessive immune activation and impaired immune responses to vaccines, but their relationships have not been examined.

**Methods:**

For selecting an aging population we enrolled 28 post-menopausal women including 12 healthy volunteers and 16 HIV-infected women on antiretroviral treatment with <100 HIV RNA copies/ml. Antibody titers to trivalent influenza vaccination given during the 2011-2012 season were determined before and 4 weeks after vaccination.

**Results:**

Seroprotective influenza antibody titers (≥1:40) were observed in 31% HIV^+^ and 58% HIV-uninfected women pre-vaccination. Following vaccination, magnitude of antibody responses and frequency of seroprotection were lower in HIV^+^ (75%) than in HIV^–^ (91%) women. Plasma IL-21, the signature cytokine of T follicular helper cells (Tfh), and CD4 T cell IL-21R were upregulated with seroconversion (≥4 fold increase in antibody titer). Post-vaccine antibody responses were inversely correlated with pre-vaccination plasma TNFα levels and with activated CD4 T cells, including activated peripheral (p)Tfh. Plasma TNFα levels were correlated with activated pTfh cells (r=0.48, p=0.02), and inversely with the post-vaccination levels of plasma IL-21 (r=-0.53, p=0.02). In vitro TNFα blockade improved the ability of CD4 T cells to produce IL-21 and of B cells to secrete immunoglobulins, and addition of exogenous IL-21 to cell cultures enhanced B cell function. Higher frequencies of activated and exhausted CD8 T and B cells were noted in HIV^+^ women, but these markers did not show a correlation with antibody responses.

**Conclusions:**

In aging HIV-infected and uninfected women, activated CD4 and pTfh cells may compromise influenza vaccine-induced antibody response, for which a mechanism of TNFα-mediated impairment of pTfh-induced IL-21 secretion is postulated. Interventions aimed at reducing chronic inflammation and immune activation in aging, HIV-infected patients may improve their response to vaccines.

## Introduction

Infectious diseases take a massive toll on the well being of both the elderly and the HIV-infected population [1,2]. The situation is particularly concerning with regard to vaccine-preventable diseases, with up to 1,000 times greater risk of death in older adults compared to vaccine-aged children [3]. Indeed, the elderly (>65 yrs) account for 90% of the >35,000 affected by annual influenza epidemics [4,5]. HIV-infected people are at a significantly higher risk than the general population at all ages for acquiring seasonal influenza infection, despite vaccination and virologic control with combination antiretroviral therapy (cART) [6-8]. Seasonal influenza vaccination is recommended for elderly as well as HIV-infected individuals to reduce influenza-related morbidity and mortality [9], but immune response to influenza vaccination is frequently impaired in both these high-risk populations [10-13]. With the considerable increase in life expectancy of HIV-infected persons with cART, and the increasing incidence of new HIV infections at older ages, it is estimated that by 2015, 50% of HIV-infected population will be ≥50 years of age [14]. Given the independent detrimental effects of aging and of HIV infection on the immune system [15-17], it is important to investigate the cumulative effects of HIV and aging on immunity, e.g. as assessed by responsiveness to seasonal influenza vaccines.

Although influenza vaccines elicit both cellular and humoral responses [18], immune protection is largely correlated with post-vaccination serum antibody (Ab) titers [19]. An essential step in the generation of vaccine induced Ab-secreting B cells is the interaction of antigen-primed B cells and T follicular helper cells (Tfh) in the germinal center reaction, where Tfh cells provide essential helper function for B cells to undergo proliferation, isotype switching and somatic hypermutation (reviewed in [20]). Tfh cells are characterized by surface expression of the CXC chemokine receptor 5 (CXCR5), that promotes their homing to lymphoid germinal centers [21], and by abundant production of the cytokine interleukin (IL)-21, that plays a major role in inducing B cell differentiation and proliferation [22] and in preserving plasma cells [23,24]. Recently, a CD4 T memory cell subset in peripheral blood bearing functional and partial phenotypic similarity to lymph node Tfh has been identified and has been designated as peripheral (p)Tfh [25-27]. The pTfh cells represent approximately 15% of circulating CD4 T cells in humans [27], express CXCR5 and provide critical help to B cells for antibody secretion in an IL-21-dependent manner [26]. In a study of vaccine responses to the pandemic H1N1/09 influenza vaccine in HIV infected young patients, impaired vaccine responses were associated with defective function of pTfh and in the IL-21/IL-21R system [27,28]. In physiologic aging, impaired Ab responses to seasonal influenza vaccination have been largely attributed to intrinsic B and T cell defects [29-33], but pTfh have not been investigated. 

A common feature of both HIV/AIDS and aging is the associated inflammation and immune activation of varying degrees [34,35], which is nevertheless greater in HIV-infected individuals than in age-matched uninfected controls, as we recently demonstrated in a cohort of post-menopausal women [36]. Tumor necrosis factor (TNF)α, one of the first pro-inflammatory factors that was described in HIV infection [37], is elevated also in the elderly [38,39], and its increased levels have been associated with disease progression and treatment failure in HIV-infected patients [40-42], and with increased risk for atherosclerosis in the aging population [43]. Intriguingly, this cytokine has been reported to decrease both T and B cell responses in vitro [44-46]. Excessive immune activation is known to favor disease progression and to hinder CD4 T cell immune reconstitution in HIV-infected persons [47-49]. In this study we have demonstrated that immune activation and the proinflammatory cytokine TNFα can have a detrimental effect on influenza vaccine responses in aging HIV-infected post-menopausal women. 

## Materials and Methods

### Study population

Women were considered eligible if they were older than 45 years of age, had been amenorrheic for ≥12 months, were not receiving hormonal replacement therapy, steroids, immunosuppressant medications, and did not have active malignancies. HIV infection was documented by licensed EIA and, if needed, confirmatory western blotting prior to enrollment. The study protocol and the informed consent forms were approved by the University of Miami Institutional Review Board and participants were enrolled after written informed consent was obtained. The study population included 12 HIV-uninfected (HIV^–^) and 16 HIV-infected (HIV^+^) women on ART with virologic suppression (HIV RNA <100 copies/ml) for ≥6 months prior, median CD4 count of 519 cells/mm^3^ (190-1,046 cells/mm^3^), and median nadir CD4 count of 124 cells/mm^3^ (4-249 cells/mm^3^). ART included two nucleoside reverse transcriptase inhibitors with either a Ritonavir boosted protease inhibitor, the non-nucleoside reverse transcriptase inhibitor Efavirenz or the integrase inhibitor Raltegravir. HIV^+^ and HIV^–^ women were matched for age and time to menopause. Characteristics of the study population are summarized in Table **S1**. 

Study participants were given a single non-adjuvanted dose of the 2011-2012 recommended influenza vaccine, containing 15 μg hemagglutinin of each of the following strains: A/California/7/2009 (H1N1), A/Perth/16/2009 (H3N2) and B/Brisbane/60/2008 (FLUARIX^®^ from GSK, UK). Vaccine preparation included the same viruses that were used for the 2010-2011 influenza season. Eighteen participants (5 HIV^–^ and 11 HIV^+^) had been vaccinated in the previous season whereas vaccination history was unavailable in the remainder. None had influenza symptoms at enrollment or follow up. 

### Processing of blood samples

Peripheral venous blood was collected by venipuncture into heparinized tubes at pre-vaccination (t0) and four weeks post-vaccination (wk4). Blood samples were processed immediately after collection. Peripheral blood mononuclear cells (PBMC) were isolated by Ficoll-hypaque density sedimentation, cryopreserved in FBS + 10% DMSO and stored in liquid nitrogen; plasma was stored at -80°C.

### Serological assessments

#### Hemagglutination inhibition assay (HIA)

Antibody titers specific for the whole vaccine were determined in t0 and wk4 plasma samples by HIA [50]. Whole vaccine was used instead of the individual antigens because the Ab titers were comparable to those seen against the individual viral strains [32]. Briefly, receptor-destroying enzyme (Denka Seiken, Campbell, CA) was added to the plasma samples to remove nonspecific inhibitors, incubated overnight at 37°C, and heat inactivated at 56°C for 1 hour. For each sample, a two-fold serial dilution was incubated with an equal volume (25 μl) of 4 hemagglutinin units of the same vaccine that was given to study participants (FLUARIX^®^, GSK) for 1 hour at room temperature. Fifty μl of 1% suspension of chicken red blood cells (Rockland™, Gilbertsville, PA) were added to each well, and antibody titers were determined 2 hours later. Paired pre- and post-vaccination samples from the same donor were tested simultaneously; each serial dilution was performed in triplicate. Seroprotection was defined as a vaccine-specific Ab titer ≥1:40, seroconversion was defined as a ≥4-fold increase in post-vaccination titers.

#### Multiplex plasma cytokine measurement

TNFα, IL-6 and IL-8 plasma levels were measured using a customized MILLIPLEX™ Cytokine Human Ultrasensitive magnetic bead panel (EMD Millipore, Billerica, MA). Undiluted samples were incubated overnight with cytokine-specific beads at 4°C with shaking. After washing, biotinylated detection antibodies and streptavidin-PE were subsequently added. The beads were washed and acquired on a MAGPIX instrument (Luminex Corporation, Austin, TX). Mean fluorescent intensity (MFI) data were collected and analyzed with MILLIPLEX™ Analyst Software (EMD Millipore). 

#### Plasma IL-21 measurement

IL-21 levels were measured in undiluted plasma samples by ELISA (eBioscience, San Diego, CA), following manufacturer’s protocol. 

### Multicolor flow cytometry

#### Monoclonal antibodies

The following anti-human monoclonal antibodies (MoAbs) were utilized for flow cytometry studies: CD3 AmCyan, Ki-67 PerCPCy5.5, CXCR5 AF647, IL-21R APC, CD20 APCCy7, CD21 PeCy5, CD27 AF700 and PD-1 PECy5 from BD Biosciences (San Jose, CA); CD4 QDot655, CD8 QDot605, CD10 QDot605 and CD45RO ECD from Invitrogen (Eugene, OR); HLA-DR AF488, CD38 PerCP, CD28 FITC, FcRL4 PE and CD57 APC from BioLegend (San Diego, CA). Live/Dead® Fixable Violet Dead Cell Stain (ViViD) Kit (Invitrogen) was used for exclusion of dead cells. Appropriate isotype control MoAbs were used for gating. 

#### Immunophenotyping

Cryopreserved PBMC were thawed and rested overnight. One million cells were incubated with ViViD stain and MoAbs for surface markers for 15 minutes, washed, fixed, permeabilized with Cytofix/Cytoperm Buffer (BD Biosciences), and stained for intracellular marker Ki-67 for 30 minutes. Cells were then washed, resuspended in PBS + 1% paraformaldehyde and acquired on a BD Biosciences LSRFortessa analyzer after proper instrument setting and compensation [51,52]. At least 500,000 events in the lymphocyte gate were acquired per sample. Data analysis was performed using FlowJo platform (TreeStar, San Carlo, CA). Frequencies of desired markers were determined in gated live (ViViD^–^) cells. T cell populations were defined as follows: CD4 (CD3^+^CD4^+^), pTfh (CD3^+^CD4^+^CD45RO^+^CXCR5^+^, as described in [26]) and CD8 (CD3^+^CD8^+^) cells. B cell differentiation subsets, determined based on phenotypic characteristics described by Moir and Fauci (reviewed in [53]) and also by us [28], were defined as: early transitional (CD21^lo/neg^CD27^–^CD10^+^), late transitional (CD21^hi^CD27^–^CD10^+^), naïve (CD21^hi^CD27^–^CD10^–^), resting memory (CD21^hi^CD27^+^CD10^–^), activated memory (CD21^lo/neg^CD27^+^CD10^–^), and exhausted tissue-like (CD21^lo/neg^CD27^–^CD10^–^).

### Memory CD4+B cell co-cultures

#### Cell enrichment and culture

B cells were isolated from healthy donor PBMC by positive selection using the EasySep™ Human CD19 Positive Selection Kit (Stemcell Technologies, Vancouver, Canada). From the remaining cells, memory (CD45RA^–^) CD4 T cells were obtained by negative selection with the EasySep™ Human Memory CD4 T Cell Enrichment Kit (Stemcell Technologies). Purity of cell populations was 95-98%. B and memory CD4 T cells were co-cultured at a 1:1 ratio in duplicate wells. The following reagents were added to the cultures: 200 ng/ml staphylococcal enterotoxin B (SEB, Sigma-Aldrich), 50 ng/ml recombinant human (rh)TNFα, 2 μg/ml anti-human TNFα blocking antibody, 50 ng/ml rhIL-21 (Invitrogen), 20 μg/ml rhIL-21R-Fc chimera (R&D Systems, Minneapolis, MN). Appropriate isotype Abs were used as controls. Culture supernatants were tested for levels of IL-21 (day 3), IgM, IgG and IgA (day 7) using commercially available ELISA kits (from eBioscience and Bethyl Laboratories, respectively). Cells were phenotyped as described above.

### Statistical analysis

Differences between groups were analyzed by Student’s *t*-test, Mann-Whitney test, or one-way ANOVA according to data distribution. Correlations between two variables were evaluated by Pearson correlation and linear regression. Analyses were performed using GraphPad Prism (GraphPad Software Inc, La Jolla, CA). P values <0.05 were considered significant. Results are presented as mean ± standard deviation.

## Results

### Response to the influenza vaccine

**Figure 1:**
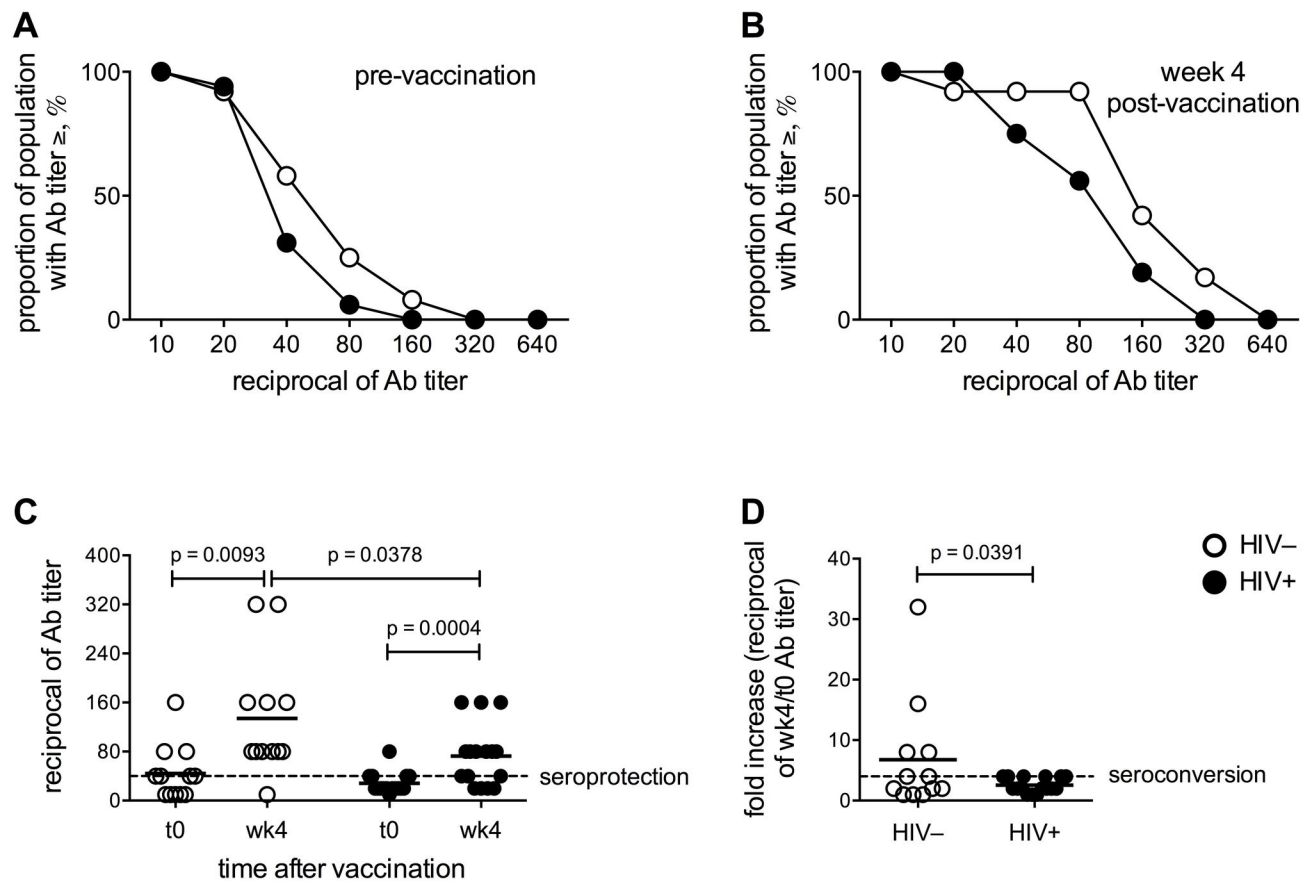
Antibody response to influenza vaccination. Antibody titers specific for the whole 2011-2012 seasonal influenza vaccine were determined before (t0) and 4 weeks (wk4) after vaccination by hemagglutination inhibition assay in the plasma of 12 HIV^–^ and 16 HIV^+^ elderly women. Reverse cumulative distribution curves for the antibody titers prior to (**A**) and 4 weeks after (**B**) vaccination for HIV-uninfected (open circles) and HIV-infected (filled circles) donors were derived to illustrate immune responses. (**C**) Reciprocal of Ab titers before and after vaccination. (**D**) Ratio between the wk4 and the baseline reciprocal of Ab titer. P values were calculated with Student’s *t*-test or Mann-Whitney test as appropriate.

The reverse cumulative distribution curves in Figure **1A and B** show anti-influenza antibody titers before and 4 weeks after vaccination, respectively. Immune responses to the influenza vaccine were overall higher in the HIV^–^ group as compared to the HIV-infected group. Before vaccination (t0), 58% of the HIV^–^ and 31% of the HIV^+^ women had a protective Ab titer (≥1:40), likely due to the persistence of antibodies generated during previous influenza seasons [54] (Figure **1A**). Four weeks after vaccination (wk4), 92% of the HIV^–^ women were seroprotected, while only 75% of the HIV^+^ women had a protective Ab titer (Figure **1B**). The increase in vaccine-specific Ab titer from baseline to wk4 post-vaccination was significant in both HIV^–^ and HIV^+^ women, but wk4 Ab titers were higher in the HIV^–^ group as compared to the HIV^+^ group (Figure **1C**). The seroconversion rate did not differ between HIV^–^ and HIV^+^ women, with 50% of the HIV^–^ and 37.5% of the HIV^+^ displaying a ≥4 fold increase of Ab titer at wk4. However, the magnitude of Ab response was significantly greater in the HIV^–^ than in the HIV^+^ women (Figure **1D**).

### Association between CD4 T cell activation and humoral response to the influenza vaccine

**Figure 2:**
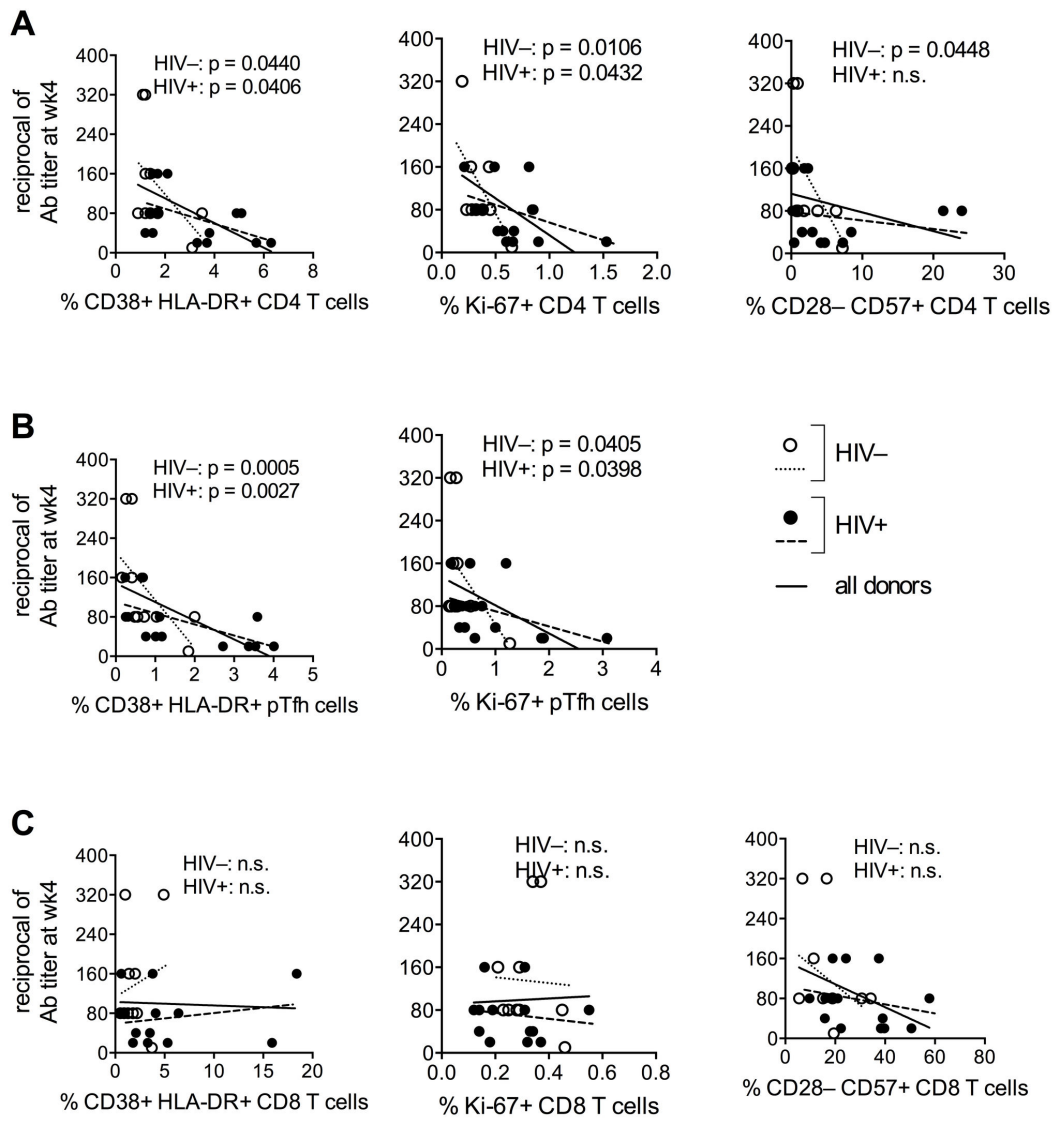
T cell activation and senescence are associated with decreased humoral response to influenza vaccination. Frozen PBMC obtained before influenza vaccination were thawed, rested over night and stained with monoclonal antibodies for immunophenotyping of CD4 (ViViD^-^CD3^+^CD4^+^), pTfh (ViViD^-^CD3^+^CD4^+^CD45RO^+^CXCR5^+^) and CD8 (ViViD^-^CD3^+^CD8^+^) T cell subsets. Correlations were established between the reciprocal of influenza Ab titer at week 4 and the frequency of activated (CD38^+^HLA-DR^+^), dividing (Ki-67^+^) and senescent (CD28^-^CD57^+^) CD4 (**A**), pTfh (**B**) and CD8 (**C**) T cells for 10 HIV^–^ (open dots) and 15 HIV^+^ (filled dots) post-menopausal women. Pearson analysis was utilized to establish statistical correlation between variables.

The immune phenotype of HIV^+^ aging women consisted of higher frequency of activated (CD38^+^HLA-DR^+^), replicating (Ki-67^+^) CD4 and pTfh cells, and higher levels of activated and senescent (CD28^–^CD57^+^) CD8 T cells, as compared to the HIV^–^ controls (Table **S2**). There was an inverse correlation between the frequency of activated CD4 and pTfh cells and the Ab titer at wk4 post-vaccination, both in the HIV^–^ and in the HIV^+^ women (Figure **2A and B**). An inverse correlation was observed between wk4 Ab titers and the frequency of senescent CD4 T lymphocytes in the uninfected group (Figure **2A**). No association was observed between CD8 T cell activation or senescence and post-vaccination Ab titers (Figure **2C**). There was no association between CD4 or pTfh activation and pre-vaccination Ab titers (not shown).

### B cell alterations in HIV^+^ women are not associated with Ab response to the influenza vaccine

**Figure 3:**
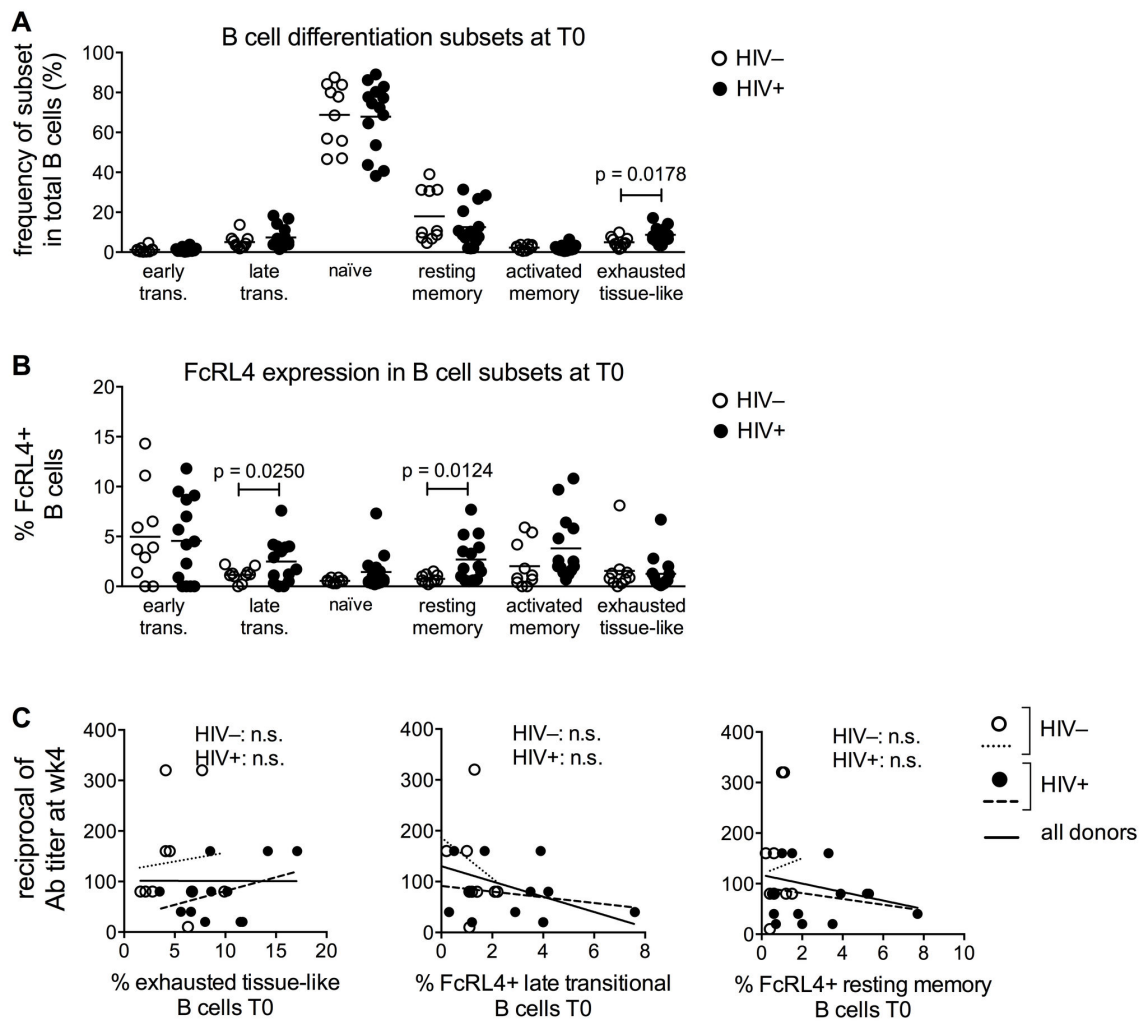
Phenotypic alterations in B cell subsets in HIV-infected aging women are not associated with antibody response to influenza vaccination. PBMC were stained with surface MoAbs to CD20, CD21, CD10, CD27 and FcRL4. Lymphocytes were gated based on forward and side scatter. CD3^-^CD20^+^ cells were gated into CD21^hi^ and CD21^lo/neg^ cells and further divided based on the expression of CD27 and CD10 as: early transitional (CD21^lo/neg^CD27^-^CD10^+^), late transitional (CD21^hi^CD27^-^CD10^+^), naïve (CD21^hi^CD27^-^CD10^–^), resting memory (CD21^hi^CD27^+^CD10^–^), activated memory (CD21^lo/neg^CD27^+^CD10^–^), and exhausted tissue-like (CD21^lo/neg^CD27^-^CD10^–^) subsets. (**A**) The percentage of cells in each B cell subpopulation was determined in 10 healthy controls (open dots) and 15 HIV-infected women (filled dots) before vaccination. (**B**) Expression of the exhaustion marker FcRL4 was evaluated in each B cell subset. (**C**) Correlations were established between the B cell populations that resulted significantly different between HIV- and HIV+ in A and B and the reciprocal of influenza Ab titer at week 4. Correlation between variables was established with Pearson analysis.

In HIV infection, B cell abnormalities in both subsets distribution and phenotype markers have been associated with altered effector function [55]. Pre-vaccination frequencies of B cell differentiation subsets determined in HIV-uninfected and infected aging women are depicted in Figure **3A**. B cell distribution into differentiation subsets in HIV^+^ patients differed from uninfected controls as previously described [28,56]. Expression of the inhibitory receptor FcRL4 was found to be higher in several B cell subsets in the HIV^+^ group as compared to the HIV^–^ controls (Figure **3B**). No significant changes were observed at wk4 post vaccination in relation to t0 (not shown). In contrast to what shown for CD4 and pTfh activation status (Figure **2A and B**), no association between B cell alterations and wk4 influenza Ab titers were observed in HIV-uninfected or infected donors (Figure **3C**).

### TNFα plasma levels inversely correlate with Ab response to the influenza vaccine, and are associated with CD4 T and pTfh cell activation

**Figure 4:**
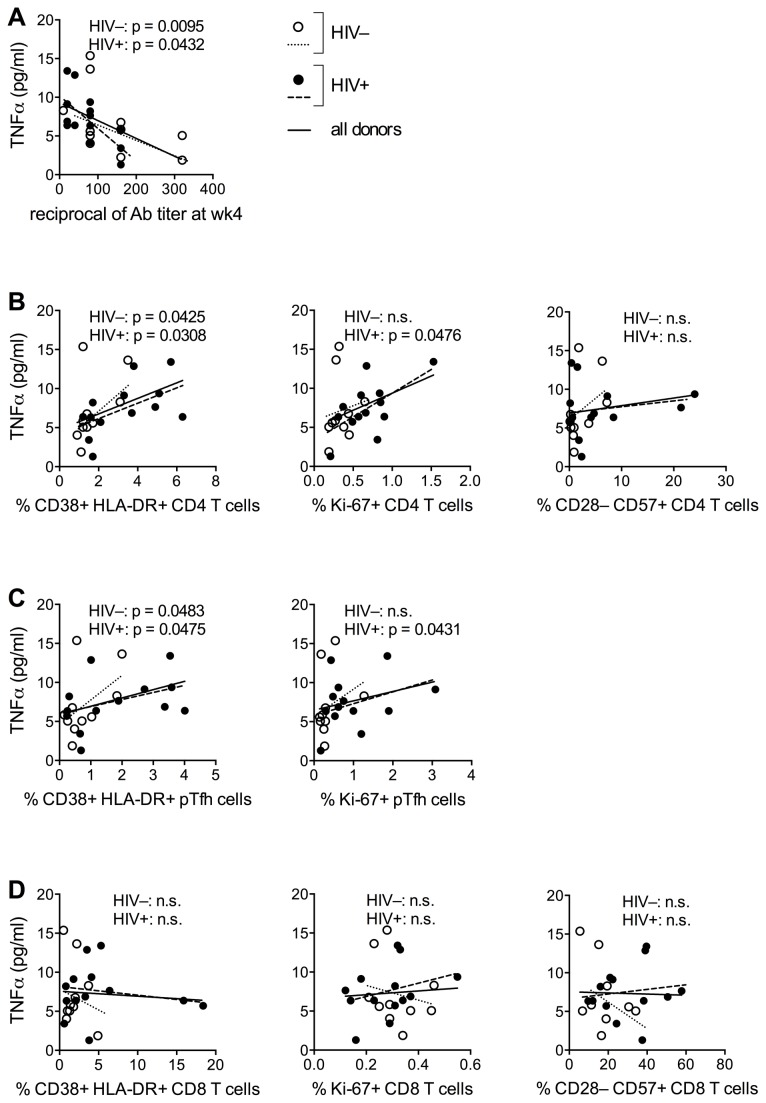
Association between circulating levels of TNFα, Ab response to influenza vaccination and T cell activation and senescence. (**A**) Correlation between TNFα plasma levels at t0 and the reciprocal of vaccine-specific Ab titers at week 4 was established for 12 HIV^–^ and 14 HIV^+^ aging women. Plasma TNFα levels were measured using a customized MILLIPLEX™ Cytokine Human Ultrasensitive magnetic bead panel (EMD Millipore). (**B**-**D**) T cells were immunophenotyped using MoAbs specific for activation (CD38, HLA-DR), proliferation (Ki-67) and senescence (CD28, CD57) markers. Frequencies of CD4, pTfh and CD8 T cell subsets expressing these markers were correlated with pre-vaccination TNFα plasma levels. Statistical analysis was performed using Pearson correlation.

We sought to investigate the relation between levels of proinflammatory cytokines TNFα, IL-6 and IL-8 (see Table **S2** for pre-vaccination levels of these cytokines) and magnitude of antibody response to the influenza vaccine. In both HIV^–^ and HIV^+^ donors, pre-vaccination plasma TNFα levels correlated inversely with wk4 Ab titers (Figure **4A**), directly with the frequency of activated and dividing CD4 and pTfh cells (Figure **4B and C**), and did not show an association with frequencies of senescent CD4 and CD8 T cells (Figure **4B and D**), or with CD8 T cell activation (Figure **4D**). No association was observed between Ab titer and IL-6 or IL-8 plasma levels, or with altered B cell subsets observed in Figure **3A and B** (not shown).

### TNFα promotes T cell activation and impairs the secretion of IL-21 and immunoglobulins in vitro

**Figure 5:**
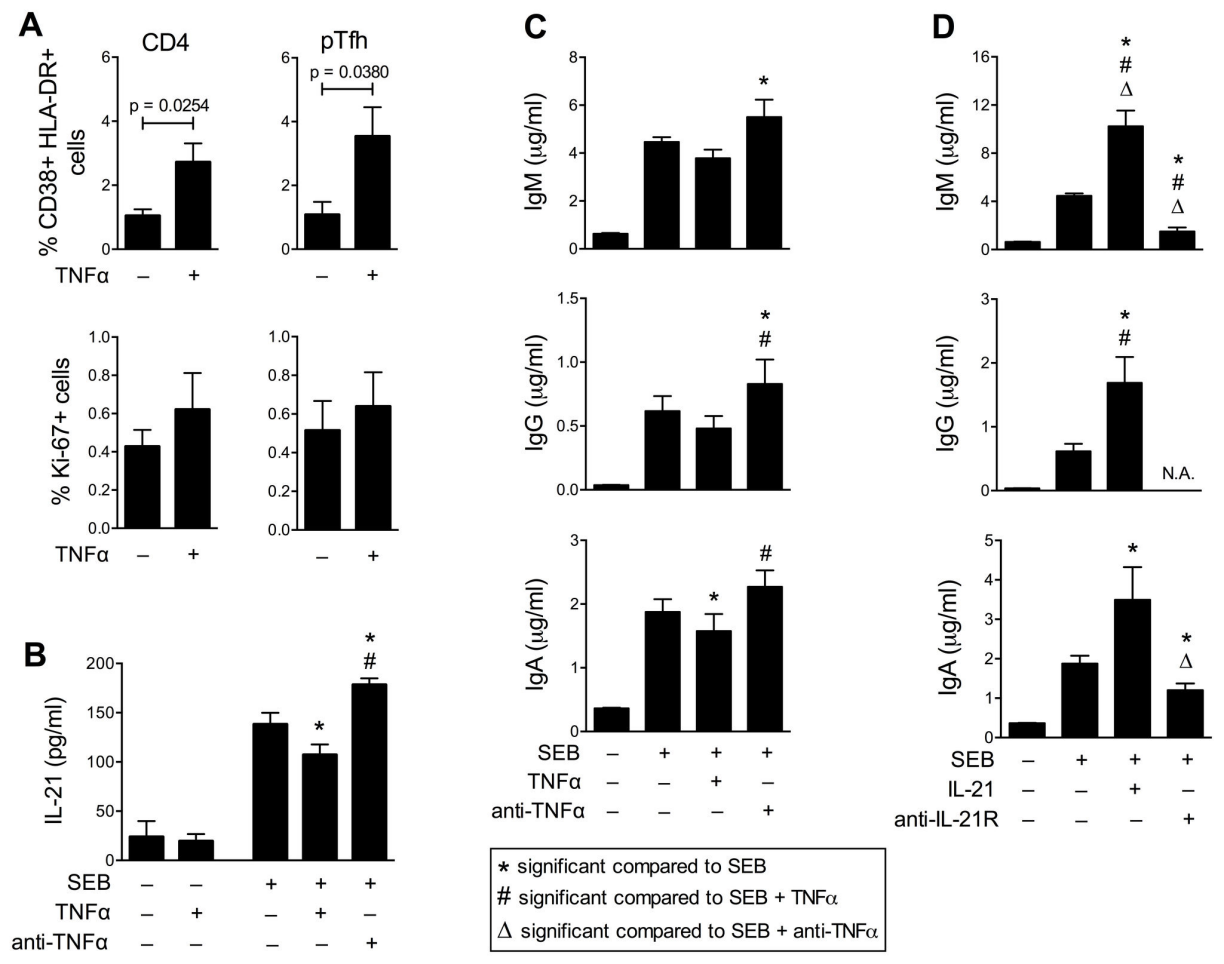
TNFα blockade promotes IL-21 secretion and IgG production in CD4-B cell co-cultures. Memory CD4 T cells obtained from 4 healthy donors were cultured with autologous B cells at a 1:1 ratio in the presence of SEB and/or TNFα, IL-21, anti-anti-TNFα or anti-IL-21R blocking antibodies or the appropriate isotype control. (**A**) Phenotyping was performed on day 3 of co-culture in gated live CD4 and pTfh cells. (**B**) IL-21 levels measured by ELISA in day 3 culture supernatants. (**C**, **D**) IgM, IgG and IgA levels were evaluated by ELISA after 7 days of co-culture. In (**D**), IgG levels in samples where IL-21R-Fc was added were not measurable because of the cross-reactivity to the Fc portion of IL-21R-Fc. P values were calculated using paired Student’s *t*-test for comparisons between two groups, and repeated measures one-way ANOVA for comparisons between larger groups.

Since high pre-vaccination plasma levels of TNFα were associated with pTfh activation status and low Ab response to the influenza vaccine (Figure **4A and C**), we sought to investigate the effect of TNFα on T and B cells in vitro. We isolated memory CD4 T cells (as surrogates of pTfh cells) from leukopacks of 4 young healthy donors [frequency of pTfh within the purified memory CD4 T cells was comparable across all donors (22.3±2.48%)] and co-cultured them with autologous B cells in the presence of SEB. As expected, the addition of exogenous TNFα increased the expression of activation markers in both CD4 and pTfh cells (Figure **5A**). Importantly, the addition of exogenous TNFα to SEB-stimulated cultures resulted in decreased IL-21 production, and maximum secretion of IL-21 was detected in the presence of blocking TNFα antibody (Figure **5B**). Moreover, in the presence of SEB, exogenous TNFα resulted in decreased immunoglobulin levels, while TNFα blockade significantly increased immunoglobulin production as compared to SEB alone (Figure **5C**). Immunoglobulin production increased in IL-21-supplemented cultures, and it was drastically reduced in the presence of IL-21R-Fc blocking Ab (Figure **5D**). When IL-21 and TNFα were simultaneously blocked, no differences in immunoglobulin levels were observed as compared to the samples in which only IL-21 was blocked (not shown).

### Changes in the IL-21/IL-21R system associated with antibody response to the influenza vaccine

**Figure 6:**
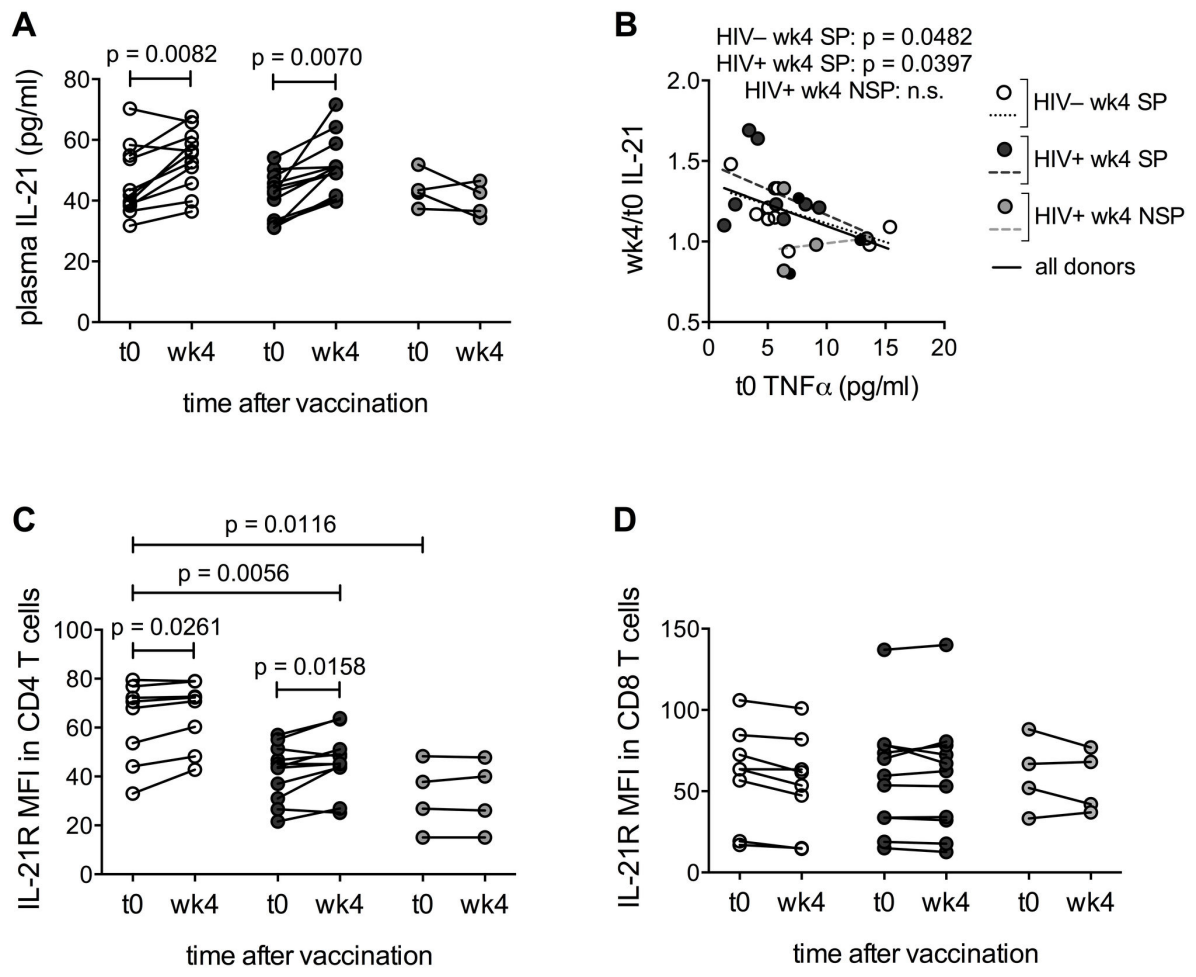
Increased plasma IL-21 and IL-21R expression in CD4 T cells is associated with humoral response to the influenza vaccine. (**A**) Circulating levels of IL-21 were measured by ELISA at t0 and wk4 in the plasma of 11 HIV^–^ seroprotected (HIV^–^ SP), 12 HIV^+^ seroprotected (HIV^+^ SP) and 4 HIV^+^ non seroprotected (HIV^+^ NSP) post-menopausal women. (**B**) Correlation between TNFα plasma levels at baseline and the ratio between wk4 and baseline plasma IL-21. (**C**, **D**) IL-21R mean fluorescence intensity (MFI) was established in live CD4 (**C**) and CD8 (**D**) T cells of 8 HIV^–^ SP, 11 HIV^+^ SP and 4 HIV^+^ NSP aging women. Frozen cells obtained before and four weeks after vaccine administration were thawed, rested overnight, stained for surface markers and acquired by flow cytometry. IL-21R MFI was established for ViViD^-^CD3^+^CD4^+^ or ViViD^-^CD3^+^CD8^+^ events. Statistical analysis was performed using paired Student’s *t*-test for comparisons within groups, unpaired Student’s *t*-test for comparisons between groups, and Pearson correlation as appropriate.

We previously observed that a protective humoral response to the influenza vaccine is associated with increased levels of IL-21 and increased expression of IL-21R in B cells post-vaccination [28]. Investigation of the IL-21/IL-21R system revealed an increase in IL-21 plasma levels from t0 to wk4 in those HIV^–^ and HIV^+^ aging women who were seroprotected at wk4, but not in the HIV^+^ non-seroprotected women (Figure **6A**). The post-vaccination plasma levels of IL-21 correlated with the wk4 Ab titers (not shown). The increase in IL-21 plasma levels from t0 to wk4 was inversely associated to the pre-vaccination circulating levels of TNFα (Figure **6B**). IL-21R MFI in CD4 T cells pre-vaccination was significantly higher in HIV^–^ as compared to the HIV^+^ women, both in seroprotected and non-seroprotected participants (Figure **6C**). An increase in IL-21R MFI from t0 to wk4 was detected only in CD4 T cells of seroprotected women, while no changes in IL-21R MFI were observed in CD4 T cells from non-seroprotected donors, or in the CD8 T cell compartment of either SP or NSP donors (Figure **6D**). IL-21R expression was also increased post-vaccination in B cells of seroprotected women, although not significantly (not shown).

## Discussion

Influenza infection is associated with significant morbidity and mortality in the elderly and HIV-infected individuals, underscoring the importance of effective vaccination-induced protection for these populations. It is well established however that both these high risk groups can have impaired influenza vaccine responses [10,13,29-33]. Our present study in post-menopausal women implies that the burden of HIV infection on top of aging is likely to increase the risk for deficient influenza vaccine responses in comparison to HIV uninfected aging women. We describe the novel association of impaired vaccine response to ongoing immune activation. In particular, we demonstrate that an activated state of peripheral T follicular helper cells and decreased vaccine-induced plasma IL-21, the signature cytokine of Tfh, are associated with impaired influenza-vaccine induced antibodies. We provide novel experimental data implicating TNFα as a major contributor for these deficiencies. 

For selecting a clear functional state of aging, we performed this study in post-menopausal women and thus avoided any influence of perimenopausal hormonal fluctuations on inflammation and immune activation. The study participants were recipients of the 2011-2012 seasonal influenza vaccine, which contained the same strains that had been included in the 2010-2011 vaccine composition [containing an A/California/7/2009 (H1N1)-like virus, an A/Perth/16/2009 (H3N2)-like virus and a B/Brisbane/60/2008-like virus]. In our study cohort, the pre-vaccination influenza Ab titers were in the protective range (≥1:40) in 58% of HIV^–^ and 31% of HIV^+^ aging women, presumably due to persisting or cross-reactive antibodies resulting from previous infection or vaccination. Although seroconversion rates were similar between HIV-infected and uninfected women, they differed in the response to vaccination. In the HIV^+^ women, antibody titers at wk4 and the fold increase in titers from t0 were lower, with several showing <4 fold increase in comparison to the HIV^–^ control group, pointing to less immune competence in the HIV-infected women.

To elucidate factors contributing to immune impairment, we investigated the relationship between Ab responses and cellular immune activation and inflammatory cytokines. Several studies have previously shown that activated CD8 T cells are associated with disease progression in HIV infection [48,49,57]. Interestingly, for the influenza vaccine-induced Ab response, it was CD4 T cell activation rather than CD8 T cell activation that showed this inverse relationship, suggesting that immune activation may have different outcomes that are specific for each cell type. Since among CD4 T cells, the pTfh subset promotes B cell proliferation and maturation [25-27], we investigated this subset and noted that the presence of activated pTfh cells prior to vaccination had a strong negative correlation with magnitude of post-vaccination Ab titers, both in HIV-uninfected and HIV-infected donors. This observation suggests that in an activated state, pTfh cells are less competent in providing the needed help to B cells to differentiate into Ab-producing cells, ultimately leading to impaired Ab response to influenza vaccine.

Both aging and HIV infection can lead to excessive inflammation. The inflammation that accompanies physiologic aging has disparate etiologies including, but not limited to, hormonal changes, diet, environmental and genetic causes. We have recently reported that in the post-menopause state several measures of immune activation and inflammation, notably T cell activation and exhaustion, monocyte/macrophage activation, and proinflammatory cytokines IL-6, IL-8 and TNFα, were higher in HIV^+^ women as compared to HIV-uninfected controls [36]. The drivers of immune activation in HIV disease in virally suppressed individuals are not well understood, but may include microbial translocation due to persistent virus-induced injury in gut-associated lymphoid tissue [58]. However, no association between markers of microbial translocation (circulating levels of LPS, monocyte/macrophage activation marker sCD14) and magnitude of antibody response to the influenza vaccine was observed in our study cohort (data not shown). Levels of plasma TNFα had a significant negative relationship with vaccine-specific Ab titers at week 4 post-vaccination in both HIV^–^ and HIV^+^ aging women. Prolonged TNFα exposure is known to impair T cell responses (namely TCR-induced proliferation and cytokine production) by attenuating T cell receptor signaling [44,45], and anti-TNF therapy in patients with psoriasis and inflammatory bowel disease results in improved responses to TCR stimulation ex vivo [59]. We investigated the influence of TNFα experimentally by adding it to cell cultures and noted that it could lead to upregulation of activation markers on CD4 T cells including pTfh cells. TNFα blockade in co-cultures of purified memory CD4 T cells and B cells enhanced Ab production supporting the contention that TNFα has a negative influence on Ab responses. Additionally, TNFα can potentially inhibit B cells directly as suggested by studies in mice [46] and by the observation that B cells of aging individuals can secrete TNFα with intrinsic inhibition of their function [60].

The helper function of Tfh and pTfh cells is mediated by cognate interaction with B cells and IL-21 secretion [25-27,55,61]. We previously demonstrated in young HIV^+^ adults who responded to influenza vaccine that their plasma IL-21 levels increased with upregulation of IL-21R in B cells, while vaccine non-responders failed to do either [28]. In the present study as well, we observed higher plasma IL-21 levels only in participants who were seroprotected at week 4, and frequency of IL-21R expression in CD4 T cells was also higher in these individuals. These findings expand the role of the IL-21/IL-21R system in regulating vaccine responses by activities not only in the B- but also in the CD4 T cell compartment. A trend of IL-21R upregulation in B cells was also observed, even though it did not reach statistical significance likely due to small sample size. Interestingly, IL-21R levels in CD8 T lymphocytes remained unaltered, implying that mechanisms other than IL-21 are involved in the generation of influenza-specific CD8 T cells. We have previously shown that the pTfh CD4 T cell subset resides almost exclusively in the memory CD4 T cell pool and that IL-21 production by pTfh is critical for influenza Ab response [27]. Here we demonstrated that addition of TNFα resulted in decreased IL-21 secretion in CD4 cell cultures, whereas IL-21 addition in our experimental model resulted in increased Ig production. As expected, IL-21 blockade resulted in suppressed Ig production based on the known critical role of IL-21 in supporting B cell function [22-24]. Taken together, our findings imply that excessive TNFα can impair pTfh function and IL-21 secretion, resulting in deficient help to B cells and poor Ab responses. Lack of clinical samples precluded us from doing experiments to investigate the mechanism of TNF alpha on adaptive immune responses in co-culture studies. We cannot exclude the possibility that in vivo, the decreases in IL-21 and antibody secretion iresulted from death of activated cells or a direct inhibitory effect of TNF-alpha on B cells. 

Previous studies have implicated immune senescence for failure to respond to influenza vaccination [62,63]. Accumulation of senescent lymphocytes has also been reported for HIV-infected individuals [64,65], possibly as consequence of chronic antigenic stimulation. We observed that senescent CD4 and CD8 T cells in our cohort were in higher frequency in HIV^+^ women. Since senescent T lymphocytes are characterized by lack of the costimulatory signal transduced by CD28, decreased ability to secrete IL-2 upon activation and poor proliferative capacity (reviewed in [66]), it is not surprising that accumulation of these cells is associated with decline of the immune function and poor humoral response to influenza vaccination. B cell exhaustion and loss of memory B cells in HIV-infected adults [67] have been associated with impaired humoral response to influenza vaccination. In our cohort alterations in B cell subsets distribution and increased B cell exhaustion (FcRL4) were observed in HIV^+^ as compared to the uninfected participants. However, no significant association between vaccine-specific Ab titers and these B cell abnormalities was observed in this cohort. Moreover, the fact that no correlation between altered B cell subsets and TNFα levels was observed suggests that the overriding defect is that of TNFα-derived excessive T cell activation.

Limitations of this pilot study are the small sample size, absence of data for other causes of immune activation such as chronic co-infections e.g. with CMV, HCV [68,69], and possibility of residual HIV replication despite virus suppression [70], and the lack of a study group composed of young HIV-infected and uninfected donors, that would have allowed to dissect the effects of aging versus HIV infection on the immune response to influenza vaccine. Moreover, Ab titers were established for the whole vaccine rather than for its three individual components, possibly underestimating the contribution of the specific strains to the seroprotection levels of the donors. Nevertheless, this is the first study to focus on influenza vaccine-induced Ab responses in the context of aging and HIV infection and immune activation. The novel finding reported herein is the association of activated pTfh and impaired IL-21 production, with negative correlation of TNFα with poor Ab responses. Elevated plasma TNFα levels are present in HIV^–^ aging population as well as in young HIV^+^ individuals [35,71]. Our results provide a rationale to further evaluate the role of pTfh in vaccine response in elderly HIV^+^ individuals and in other proinflammatory conditions where abnormal antibody responses are observed upon vaccination. Interventions aimed to reduce chronic inflammation and activation in HIV-infected patients may improve their response to vaccination. Studies in larger cohorts may help to define predictive immunologic signatures of vaccine responses.

## Supporting Information

Table S1 **Characteristics of the study population.** Demographic characteristics and HIV disease markers of the study population. Menopause was defined as lack of menstruation for more than 12 months. Median values are shown for age, time to menopause and CD4 counts. Lower limit for plasma HIV RNA detection was 20 copies/ml. Statistical differences between groups were analyzed by Student *t*-test.
(PDF)

Table S2 **Cellular and soluble markers of immune activation, exhaustion and senescence in HIV- and HIV+ aging women.** Cryopreserved PBMC were thawed and rested overnight, stained with ViViD and monoclonal antibodies and acquired on a flow cytometer. Lymphocytes were gated based on forward and side scatter, and gates for exclusion of singlets and dead cells (ViViD+ events) were drawn. Expression of activation (CD38, HLA-DR, Ki-67), exhaustion (PD-1) and senescence (CD28, CD57) markers was evaluated in live CD4, pTfh and CD8 T cells. Plasma levels of cytokines were measured using a customized MILLIPLEXTM Cytokine Human Ultrasensitive magnetic bead panel (EMD Millipore). Statistical differences between groups were analyzed by Student *t*-test. Significant P values are shown in bold.
(PDF)
