# Supplementary material for: Impaired Antibody Response to Influenza Vaccine in HIV-Infected and Uninfected Aging Women Is Associated with Immune Activation and Inflammation
Source: PLoS One. 2013 Nov 13;8(11):e79816. doi: 10.1371/journal.pone.0079816 (PMC3827419; doi:10.1371/journal.pone.0079816)
Supplement: Table S1 — Characteristics of the study population. Demographic characteristics and HIV disease markers of the study population. Menopause was defined as lack of menstruation for more than 12 months. Median values are shown for age, time to menopause and CD4 counts. Lower limit for plasma HIV RNA detection was 20 copies/ml. Statistical differences between groups were analyzed by Student t-test. (PDF) [file pone.0079816.s001.pdf]

Supplemental Table S1: Characteristics of the study population

	HIV^- women<br>N = 12	HIV^+ women<br>N = 16	p
**Demographics**			
Age (years)	57 (53-65)	55 (49-66)	0.2567
Time to menopause (years)	13 (3-18)	10 (2-20)	0.2454
**Features of HIV infection**			
CD4 cell count (cells/mm^3)		519 (190-1,046)	
CD4 nadir count (cells/mm^3)		124 (4-249)	
HIV RNA (copies/ml)		undetectable-80	
