# Supplementary material for: Impaired Antibody Response to Influenza Vaccine in HIV-Infected and Uninfected Aging Women Is Associated with Immune Activation and Inflammation
Source: PLoS One. 2013 Nov 13;8(11):e79816. doi: 10.1371/journal.pone.0079816 (PMC3827419; doi:10.1371/journal.pone.0079816)
Supplement: Table S2 — Cellular and soluble markers of immune activation, exhaustion and senescence in HIV- and HIV+ aging women. Cryopreserved PBMC were thawed and rested overnight, stained with ViViD and monoclonal antibodies and acquired on a flow cytometer. Lymphocytes were gated based on forward and side scatter, and gates for exclusion of singlets and dead cells (ViViD+ events) were drawn. Expression of activation (CD38, HLA-DR, Ki-67), exhaustion (PD-1) and senescence (CD28, CD57) markers was evaluated in live CD4, pTfh and CD8 T cells. Plasma levels of cytokines were measured using a customized MILLIPLEXTM Cytokine Human Ultrasensitive magnetic bead panel (EMD Millipore). Statistical differences between groups were analyzed by Student t-test. Significant P values are shown in bold. (PDF) [file pone.0079816.s002.pdf]

Supplemental Table S2: Cellular and soluble markers of immune activation, exhaustion and senescence in HIV^- and HIV^+ aging women

	HIV^- women<br>N = 12	HIV^+ women<br>N = 16	p
**T cell activation**			
$CD38^+HLA\text{-}DR^+$ CD4 (%)	1.67±0.28	3.03±0.45	**0.0348**
$CD38^+HLA\text{-}DR^+$ pTfh (%)	0.78±0.54	1.64±1.24	0.0502
$CD38^+HLA\text{-}DR^+$ CD8 (%)	1.97±0.43	6.54±2.21	0.0592
$Ki\text{-}67^+$ CD4 (%)	0.34±0.14	0.65±0.32	**0.0102**
$Ki\text{-}67^+$ pTfh (%)	0.36±0.11	0.91±0.70	**0.0428**
$Ki\text{-}67^+$ CD8 (%)	0.31±0.09	0.36±0.12	0.2833
**T cell exhaustion**			
$PD\text{-}1^+$ CD4 (%)	14.22±6.52	26.12±12.47	**0.0111**
$PD\text{-}1^+$ pTfh (%)	21.58±7.04	42.93±20.40	**0.0043**
$PD\text{-}1^+$ CD8 (%)	18.27±10.18	21.14±8.42	0.1795
**T cell senescence**			
$CD28^-CD57^+$ CD4 (%)	2.22±2.61	5.35±7.05	0.2195
$CD28^-CD57^+$ CD8 (%)	17.60±9.74	29.23±14.50	**0.0470**
**Proinflammatory cytokines**			
TNFα (pg/ml)	6.08±3.77	7.80±3.58	0.0620
IL-6 (pg/ml)	0.96±0.68	1.46±1.64	0.3592
IL-8 (pg/ml)	3.74±1.76	4.86±2.32	0.2122
